# GLP-1 receptor agonists in osteoarthritis and psoriatic disease: the missing link between obesity and inflammation?

**DOI:** 10.1007/s00296-026-06258-2

**Published:** 2026-07-22

**Authors:** Andreas Angelopoulos, George E. Fragoulis, Charalampos Papagoras, Dimitrios Daoussis

**Affiliations:** 1 University of Patras Medical School, Patras, Greece; 2 First Department of Propaedeutic and Internal Medicine, National and Kapodistrian University of Athens, Athens, Greece; 3 First Department of Internal Medicine, University Hospital of Alexandroupolis, Democritus University of Thrace, Alexandroupolis, Greece; 4 Department of Rheumatology, Patras University Hospital, University of Patras Medical School, Patras, Greece

**Keywords:** Glucagon-Like Peptide-1 Receptor Agonists, Glucagon-Like peptide, Osteoarthritis, Arthritis, Psoriatic, Psoriasis, Obesity

## Abstract

Excessive body weight stands out as a major, well-documented risk factor for rheumatic and musculoskeletal diseases (RMDs). Indeed, high body mass index (BMI) affects disease severity, treatment response, and long-term outcomes. Glucagon-like peptide-1 receptor agonists (GLP-1 RAs) were first developed for type 2 diabetes and obesity, but there is growing evidence that they may also have anti-inflammatory and immunomodulatory properties [1–3]. This narrative review examines the available evidence on the role of GLP-1 RAs in two conditions that are strongly associated with metabolic and mechanical factors but sit on almost opposite ends of the inflammatory spectrum: osteoarthritis (OA), a predominantly mechanically driven disease, and psoriatic disease (PsD), an immune-mediated inflammatory disease. A comprehensive narrative review of the literature was conducted to evaluate preclinical and clinical evidence regarding the effects of GLP-1 signaling in both OA and PsD. Preclinical data suggest that GLP-1 signaling may protect cartilage, reduce inflammation, and alleviate pain in OA. Early clinical evidence is encouraging, showing reductions in both joint pain and body weight. In PsD, obesity and psoriatic inflammation share several common pathways, particularly through the IL-17/IL-23 axis, which provides a theoretical biological rationale for GLP-1 RAs use in this setting, although direct clinical evidence remains limited and largely derived from studies in obese patients. Dedicated randomized controlled trials are necessary to clarify the direct immunomodulatory mechanisms involved and to define the precise position of GLP-1 RAs in rheumatological practice.

## Introduction

Obesity affects approximately one in six adults globally (over a billion individuals), with projections suggesting that by 2035, more than half of the world’s population is expected to be obese [1, 2]. Obesity is an established independent factor that raises the risk of illness and early death [3, 4]. In this context, it significantly modulates the prevalence, severity, and treatment response of many rheumatic and musculoskeletal diseases (RMDs) [5]. For example, in rheumatoid arthritis and psoriatic arthritis (PsA), obesity is associated with higher disease activity, attenuated efficacy of disease-modifying anti-rheumatic drugs (DMARDs) and biologics, and lower remission rates [5]. 

Among the established risk factors for hip and knee osteoarthritis (OA) are age, female sex, genetic predisposition, prior joint injury, cardiometabolic comorbidities, and obesity [6, 7], the latter accelerating cartilage degeneration through both mechanical and metabolic mechanisms [5]. Importantly, the interplay between mechanical load and systemic inflammation in obese patients makes it difficult to distinguish metabolic from immune-mediated drivers of joint damage [8]. 

Obesity is roughly twice as common in psoriasis (PsO) patients in comparison with the general population [9]. A similar trend is evident in PsA; population-based evidence from Norway indicates a graded increase in risk (RR 1.4 and 2.5) corresponding to overweight and obese status, respectively [10]. Similar results are confirmed by two large-scale cohort studies which collectively included approximately 165,000 individuals [11, 12]. Thus, an association likely exists between fat accumulation and psoriatic disease (PsD), with obesity acting as both a pathogenic driver of disease onset and a modifier of its clinical course [10]. 

Several reviews have examined the role of GLP-1 RAs in rheumatic diseases predominantly focusing on a single condition [13, 14]. Given the relevance of the obesity-inflammation interplay in RMDs, this review examines two conditions that represent contrasting but complementary paradigms through which the obesity-inflammation axis operates. OA is the most prevalent arthritis worldwide and a classic example of a condition in which metabolic and mechanical mechanisms predominate, with low-grade inflammation playing a modulatory rather than a primary role. PsD, by contrast, is a prototypical immune-mediated inflammatory disorder, in which obesity acts as a potent amplifier of the underlying inflammatory pathways. This approach allows for a more in-depth understanding of whether the proposed effects of GLP-1 RAs in rheumatology are condition-specific or reflect a broader immunometabolic action.

Glucagon-like peptide-1 (GLP-1) functions as an incretin hormone essential for sustaining glucose homeostasis [5]. It exerts its effects by promoting insulin secretion, reducing glucagon release, and modulating gastric motility, thereby regulating postprandial glycemic excursions [5]. Except for their effects on blood sugar and metabolism, GLP-1 receptor agonists (RAs) have shown possible anti-inflammatory effects that extend beyond weight loss alone [6]. Consequently, there is a growing interest in exploring their therapeutic potential within OA and PsD.

## Methods

We performed an electronic search on Medline/Pubmed, Scopus, Web of Science, and the Directory of Open Access Journals (DOAJ) using the following MeSH terms and free-text keywords were used in Boolean combinations: (“Glucagon-Like Peptide-1 Receptor Agonists” OR “Glucagon-Like peptide”) AND (“Osteoarthritis” OR “Arthritis, Psoriatic” OR “Psoriasis”) AND (“Obesity”). Our main goal was to explore the available evidence on the role of GLP-1 RAs in two conditions where obesity plays a crucial role: OA and PsD. Our research was carried out from February 2026 to July 2026, and no date restrictions were applied. Only articles in the English language were included. Studies reporting preclinical and clinical data on the role of GLP-1 RAs in OA and/or PsD were considered eligible. Review articles, case reports and original research studies were all included where relevant. Articles not related to the topic were excluded. Additional references were identified through manual screening of the reference list of retrieved articles.

## GLP-1 biology

First identified in the 1980s, GLP-1 is produced by enteroendocrine L cells that are found mainly in the distal small intestine and colon [6]. Following nutrient ingestion, GLP-1 is quickly secreted into the bloodstream together with glucose-dependent insulinotropic polypeptide (GIP), the two principal incretin hormones that augment postprandial insulin secretion by the pancreas [6]. However, endogenous GLP-1 has a very brief half-life of nearly two minutes, with only 10%-15% of the active molecule entering the systemic circulation intact, as it is swiftly broken down by the enzyme dipeptidyl peptidase-4 (DPP-4) [5, 6]. GLP-1 RAs are synthetic compounds structurally modified to withstand DPP-4 degradation, leading to a markedly extended circulating half-life than the native hormone [5, 6]. 

The principal role of GLP-1 is glucose homeostasis and metabolic regulation [6]. Beyond stimulating insulin secretion, it suppresses glucagon release, delays gastric emptying, and enhances feelings of fullness, collectively reducing food intake and postprandial glycaemia [6]. GIP also plays a dual role by regulating glycaemia through glucose-dependent insulin secretion, while simultaneously targeting central satiety pathways and adipose tissue to drive body weight reduction [15]. Remarkably, obese individuals present lower GLP-1 response following glucose ingestion compared to lean controls, suggesting that impaired incretin signaling may contribute to the metabolic dysregulation observed in obesity [1]. 

The GLP-1 receptor (GLP-1R) is a member of the class B family of G-protein-coupled receptors and is found in many tissues, consistent with the diverse physiological roles of GLP-1 beyond glucose regulation [5, 16]. It is expressed in the gastrointestinal tract, cardiovascular system, kidneys, pancreas, brain, adipose tissue, and skeletal muscle [5, 16]. Of relevance to rheumatology, GLP-1R has also been identified on monocytes, dendritic cells, T cells and B cells, macrophages, fibroblast-like synoviocytes, along with bone marrow stem cells, osteocytes, osteoblasts, osteoclasts, chondrocytes, and has even been detected within the synovial fluid [5, 6, 17]. This wide immunological and articular expression provides a biological rationale for the potential role of GLP-1 RAs in musculoskeletal and inflammatory diseases.

Several GLP-1 RAs are presently available in clinical practice. Among those approved for type 2 diabetes (T2D) mellitus, dulaglutide, liraglutide, exenatide, and semaglutide are the most widely used [1]. Liraglutide and semaglutide carry additional approval for the management of obesity. Tirzepatide, which acts as a dual GIP/GLP-1 receptor agonist, represents a newer generation of incretin-based therapies and has demonstrated greater weight reduction than selective GLP-1 RAs [7]. Several additional agents within this class are under development, with numerous ongoing trials underway to evaluate their efficacy and safety for weight management and beyond.

## Obesity and the musculoskeletal system: the pathophysiological link

Obesity affects the musculoskeletal system primarily through two key mechanisms that frequently interact. On the one hand, excess body weight increases biomechanical stress on weight-bearing joints, particularly the joints of the knees, causing entheseal microtrauma and local inflammation [1, 7, 18]. On the other hand, obesity is closely linked to chronic systemic inflammation, where increased body weight and inflammatory activity amplify one another [19, 20]. The proportion between these two mechanisms is not always easy to distinguish, and in clinical practice, they frequently coexist.

Central to this inflammatory process is adipose tissue, which functionally is much more than a simple energy reservoir. It operates as a dynamic endocrine organ that actively secretes adipokines and a diverse array of pro-inflammatory mediators [21, 22]. This concept was first described by Ogston and McAndrew in 1964, who noted a correlation between obesity and inflammation [23]. Obesity provokes a systemic, low-grade, metabolically driven inflammatory state, called “metaflammation”, originating from dysfunctional metabolic cells, such as adipocytes, which may lower the threshold for rheumatic disease development in genetically predisposed individuals [24, 25]. 

More specifically, adipose tissue releases pro-inflammatory adipokines such as leptin, tumor necrosis factor-alpha (TNF-α), interleukin (IL)-6, and IL-18, while simultaneously suppressing anti-inflammatory mediators [26, 27]. Leptin promotes macrophage polarization toward the pro-inflammatory M1 phenotype and stimulates the production of cytokines like IL-1β, IL-6, IL-12, IL-17, and TNF-α, while concurrently reducing the activity of anti-inflammatory mediators such as transforming growth factor-beta (TGF-β) and IL-10 [26, 27]. In contrast, adiponectin, another adipokine, is generally considered to have anti-inflammatory effects [27]. Of particular relevance to OA, infrapatellar fat pads – intra-articular adipose tissue deposits present in the knee – represent a local source of adipokines and fatty acids that may contribute to the development of the knee OA (KOA) [6]. However, it is still unconfirmed whether fat pads express GLP-1R [6]. 

Obese individuals often exhibit heightened pain sensitivity compared to their normal-weight counterparts [28, 29]. This phenomenon is driven by a multifactorial process involving mechanical loading, systemic release of adipokines, and the neurotrophic priming of both peripheral and central sensitization pathways [28, 29]. Each additional unit of body mass index (BMI) correlates with a 1.5 times higher risk of developing OA [1], and weight loss has been proven to ameliorate OA symptoms and reduce joint pain, which is consistently more severe in obese patients with OA than in those without obesity [1, 18]. 

The relationship between obesity and PsD deserves particular attention. It is well established that obese individuals are at a greater risk of developing PsO and PsA [30, 31]. Metabolic syndrome occurs more frequently in PsA patients (46%) than in PsO patients (34%), and an increased BMI correlates with more severe skin disease [30, 31]. Notably, data from 2.1 million study individuals – including 200,000 PsO patients – demonstrate a markedly higher rate of obesity within this population [32]. Moreover, childhood obesity has been positively correlated with subsequent development of PsO and PsA in adulthood [33, 34]; furthermore, clinical data suggest that metabolic bariatric surgery improves outcomes and lowers the necessity for intensive pharmacological interventions [35]. 

Although the first-line approach to obesity management in patients with RMDs is lifestyle modification through exercise and proper diet, this is not always applicable to all patients for many reasons. The underlying RMD itself frequently imposes significant functional limitations and pain, creating considerable barriers to physical activity [26]. Furthermore, comorbidities such as depression, which is more prevalent in this population, may promote emotional eating and further undermine dietary adherence, complicating weight management [36]. 

## GLP-1 RAs in osteoarthritis (OA)

OA remains the most prevalent form of arthritis globally, affecting around 500 million individuals [7]. It is a main cause of functional impairment, especially in older people, affecting numerous joints such as the hips, knees, spine, and hands [3], and is marked by a gradual decline of the articular cartilage, synovial membrane alterations, and subchondral bone changes, eventually causing joint damage [37]. Clinical presentation includes joint swelling, effusion, stiffness, and pain that lead to physical inactivity and a sedentary lifestyle, further increasing the risk of cardiometabolic comorbidities and mortality [7, 37]. Current treatments remain symptomatic, as no disease-modifying OA drugs (DMOADs) are available. These include paracetamol, topical and oral nonsteroidal anti-inflammatory drugs (NSAIDs), glucosamine, chondroitin, opioid analgesics, intra-articular hyaluronates, intra-articular corticosteroid injections, and duloxetine. NSAIDs carry significant risks in older people with comorbidities, while opioid analgesics raise concerns regarding toxicity and addiction. Other treatment options include weight loss, physical therapy, supportive devices, and surgery [7, 18, 38, 39]. 

Chronic low-grade inflammation is central to OA pathogenesis, promoting progressive cartilage destruction, accelerating structural joint deterioration, and contributing to both neuronal sensitization and tissue injury [40]. Pro-inflammatory cytokines such as IL-1β, IL-6, IL-17, and TNF-α, are elevated in OA, with activation of the NF-κB pathway and macrophage polarization toward the pro-inflammatory M1 phenotype in the synovium [6, 7, 41]. Mechanical stress also promotes the secretion of catabolic enzymes – including matrix metalloproteinases (MMPs) by macrophages, a disintegrin and metalloproteinase with thrombospondin motifs (ADAMTS) by chondrocytes – as well as reactive oxygen species (ROS), resulting in progressive joint deterioration and chronic pain due to neurodegenerative changes [6, 7]. Of note, GLP-1R expression has been detected in chondrocytes within human OA knee joints, including the articular cartilage and synovial membrane [1, 42]. Additionally, OA patients have lower circulating GLP-1 levels than healthy individuals, indicating a potential role of impaired GLP-1 signaling in the development of the disease [1, 42]. 

### Evidence from animal models

Preclinical studies have offered valuable insights into how GLP-1 signaling may influence OA. Immunohistochemical analysis of healthy murine knee joints revealed GLP-1R expression in the tibial and femoral articular cartilage, meniscal tissue, and bone marrow [42]. Intra-articular injection of liraglutide has pain-relieving and anti-inflammatory effects in a short-term sodium monoiodoacetate (MIA) murine model of OA, and analgesic effect in a long-term MIA model, in the latter case by improving the synovitis severity [42]. In fact, in the long-term model, dexamethasone proved to be inferior to liraglutide in pain management. The study demonstrated that liraglutide has anti-inflammatory effects on murine chondrocytes and macrophages in vitro via GLP-1R-mediated signaling, shifting macrophage polarization away from the M1 pro-inflammatory state toward the M2 anti-inflammatory state [42]. These findings are also supported by another study showing that GLP-1 RAs shift macrophage polarization towards a less inflammatory phenotype by reducing M1 and promoting M2 macrophage differentiation [43]. 

Further experimental data from both cell cultures and rodent models suggest that GLP-1 receptor agonism exerts neuroprotective effects by stimulating neuronal proliferation, reducing neuroinflammation, and ultimately improving pain sensitivity, potentially via stimulating the release of β-endorphins and inhibiting neuronal apoptosis [44–46]. Tong et al. demonstrated exenatide’s ability to decrease the production of advanced glycation end-products (AGEs) and inhibit the activation of NF-κB in chondrocytes, providing an additional mechanistic rationale for its potential chondroprotective role [47]. Crucially, the latest data demonstrate that semaglutide exerts chondroprotective and analgesic effects through a weight loss-independent mechanism in OA mouse models. By reprogramming the metabolic profile of chondrocytes from glycolysis to oxidative phosphorylation under inflammatory conditions, semaglutide may restore cartilage integrity and reduce synovial lesions [48]. 

### Clinical evidence

Weight loss remains fundamental in OA management, reducing mechanical load on the joints and improving articular cartilage integrity, with decreasing BMI also associated with a reduced need for total knee replacement [7, 49]. Clinical studies evaluating the direct impact of GLP-1 RAs on OA have yielded largely, though not uniformly, encouraging results.

More specifically, two studies conducted in a small group of patients with obesity and T2D found that exenatide and liraglutide reduced levels of ROS, NF-κΒ, the inflammatory macrophage activation marker sCD163, as well as pro-inflammatory cytokines such as TNF-α, IL-1β, and IL-6 [50, 51]. Importantly, these effects occurred independently of body weight changes, indicating that GLP-1 RAs exert a direct anti-inflammatory effect beyond their metabolic effects [50, 51]. These findings are reinforced by a meta-analysis of 40 randomized-controlled trials comprising 6,949 individuals, which demonstrated that GLP-1 RAs significantly lower levels of C-reactive protein, TNF-α, and malondialdehyde [52]. 

In a randomized controlled trial, KOA patients who received liraglutide treatment experienced more weight loss than those in the placebo group, although no notable difference in OA pain was found between the groups [53]. In contrast, a large observational study by Zhu et al. evaluating more than 40,000 participants with KOA found that the majority of patients treated with GLP-1 RAs achieved weight reduction of at least 5%, and these patients also showed a reduced rate of knee surgery. This study also showed that the rate at which cartilage deteriorates in the medial femorotibial joint was notably slower among GLP-1 RAs users, who additionally needed fewer intra-articular corticosteroid injections [54]. 

The STEP-9 trial provides the strongest clinical evidence so far supporting the role of GLP-1 RAs in KOA [13]. This was a 68-week, double-blind, randomized, placebo-controlled trial, enrolling 407 obese participants with moderate KOA and at least moderate pain. Participants were randomly divided in a 2:1 ratio to receive either once-weekly subcutaneous semaglutide 2.4 mg or a placebo. Both groups were counseled on physical activity and a lower-calorie diet [13]. The two main outcomes measured were the percentage change in body weight and the change in the Western Ontario and McMaster Universities Osteoarthritis Index (WOMAC) pain score from baseline to week 68. Semaglutide demonstrated superiority over placebo on both primary endpoints: mean body weight reduction was 13.7% versus 3.2% with placebo (*p* < 0.001), and mean WOMAC pain score reduction was 41.7 points versus 27.5 points with placebo (*p* < 0.001) [13]. Individuals on semaglutide experienced a more significant improvement in the Short Form-36 (SF-36) physical function score, with an average increase of 12.0 points compared to 6.5 points (*p* < 0.001). They also showed a greater reduction in analgesic use, suggesting that pain relief was not attributable to pain medication [13]. The benefit in pain reduction was consistent across all BMI subgroups. The trial was not designed to investigate mechanisms, and weight reduction is likely the primary contributor through reduced mechanical loading. However, the authors acknowledge that preclinical data support direct anti-inflammatory and anti-degradative features of GLP-1 RAs, leaving open the question of weight-independent effects. Limitations include the absence of imaging follow-up, lack of inflammatory biomarker assessment, and the predominance of women (81.6%) among participants [13]. 

However, not all data available support a beneficial role for GLP-1 RAs in OA. Two studies reported a higher frequency of hip and knee OA incidence or progression among GLP-1 RA users compared to non-users, but these results are limited by their observational nature and the possibility of confounding by indication [55, 56]. Of note, isolated case reports have described the onset of inflammatory arthritis — such as polyarthritis and new-onset rheumatoid arthritis — after starting GLP-1 RA treatment, highlighting the need for vigilance regarding unexpected musculoskeletal side effects in patients treated with these agents [57, 58]. 

Overall, the available evidence indicates that GLP-1 RAs may provide benefits in OA, primarily through weight loss, with preclinical data additionally supporting possible direct chondroprotective, anti-inflammatory, and analgesic effects. However, the relative contribution of direct versus indirect mechanisms remains still unclear [7]. Possible direct effects include reduction of oxidative stress, immunomodulation, attenuation of cartilage degradation, enhancement of DNA repair, neuroprotection, and analgesia [7]. Clinical trials specifically designed for OA patients who do not have obesity will be needed to determine whether GLP-1 RAs have genuine disease-modifying potential in this condition.

## GLP-1 RAs in psoriatic disease

PsO and PsA are the major subsets of PsD that share common pathogenic mechanisms but differ in their predominant clinical expression. PsO is a chronic immune-mediated skin disease affecting 2%-3% of the global population, while PsA is an inflammatory arthritis characterized by joint and entheseal pain, stiffness, and swelling, occurring in 10%-30% of PsO patients [8, 59, 60]. The pathogenesis of PsD is complex, involving genetic, metabolic, mechanical, and microbial factors [61]. Treatment options for PsA include conventional synthetic DMARDs, biological DMARDs, such as anti-TNF, anti-IL-17, anti-IL-23, anti-IL-12/23 agents, and JAK inhibitors [62]. Similar biological agents are used for the systemic management of PsO [63]. 

The impact of obesity in this context cannot be overstated. Far from being a simple comorbidity [64], obesity acts as a potent modifier of the psoriatic phenotype, as it often leads to a heavier disease burden and a poor response to standard therapies [65–67]. Patients with high BMI have a reduced likelihood of attaining minimal disease activity (MDA) and measurably lower quality of life [65]. A marked imbalance in adipokine secretion has been documented in the psoriatic population, with studies consistently reporting low adiponectin and high leptin concentrations relative to healthy controls [27]. Presumably, the inflammatory pathways activated in obesity and PsD exhibit considerable overlap, as they share several key cytokines, such as IL-2, IL-6, IL-17, IL-23, TNF-α, and IFN-γ, which drive the pathogenesis of both conditions. Specifically regarding the IL-17/23 axis, IL-23 stimulates the differentiation of Th17 cells to induce IL-17 production, driving an inflammatory cascade in skin and joints that is further amplified in the presence of obesity [26, 68–70]. This overlap may provide a rationale for targeting obesity as part of the therapeutic strategy in PsD.

The significance of obesity on treatment results in PsA is widely recognized. Two studies, which collectively evaluated around 90 subjects, concluded that diet-induced weight loss is linked to a positive impact on disease outcomes in patients with both PsA and obesity [65, 71]. A 2014 prospective study found that a ≥ 5% body reduction was an independent predictor of achieving MDA in overweight or obese patients with PsA initiating anti-TNF-α therapy [72]. A following systematic review and meta-analysis showed that excess body weight negatively affects response to anti-TNF agents, with obese patients carrying a 60% greater likelihood of treatment failure compared to normal-weight individuals, a finding partly explained by increased volume of distribution and accelerated drug clearance [73]. Furthermore, it is noteworthy that the use of anti-TNF agents themselves is associated with a mild weight gain, which may further complicate long-term disease management [74]. In contrast, secukinumab and ixekizumab have demonstrated maintained efficacy across BMI categories in PsA, with drug retention rates and clinical response rates largely independent of body weight, although dose optimization – such as more frequent secukinumab dosing in patients weighing 90 kg or more – may be required to achieve optimal skin responses in heavier patients with PsO [75–79]. Likewise, IL-23 inhibitors seem to maintain their efficacy in obese patients with PsA [80]. 

### Evidence from animal models

Preclinical studies have shed light on the mechanisms by which obesity amplifies psoriatic inflammation. It is well established that a high-fat diet (HFD) induces inflammatory skin lesions in murine models [81]. Research in this context identified a specific subcategory of skin regulatory cells (Tregs) expressing the nuclear receptor peroxisome proliferation-activated receptor gamma (PPAR-γ). These cells mainly serve to restrict excessive psoriatic inflammation driven by IL-17 A-positive γδ T cells. Their numbers decreased after HFD, while their restoration led to attenuation of IL-17-mediated inflammation and improvement of skin psoriasis [82]. Nakamizo et al. consistently found that HFD induces the aggregation of IL-17 A-producing γδ T cells in both the skin and systemically, which worsens psoriatic dermatitis in murine models [83]. Except for IL-17, IL-23 is essential in the pathophysiology of PsD. Another study found that under conditions of acute inflammation, elevated levels of free fatty acids released by the adipose tissue enhance the secretion of IL-23 and IL-6 from dendritic cells. Specifically, these fatty acids initiate a cellular stress reaction through the accumulation of mitochondrial ROS, which in turn activates the unfolded protein response [84]. This process ultimately amplifies the inflammatory response, driving the epidermal thickening and skin damage typically seen in PsO [84]. These findings collectively illustrate the mechanisms through which obesity fuels psoriatic inflammation and suggest potential targets for therapeutic intervention.

### Clinical evidence

Weight loss has been shown to improve PsO symptoms and Psoriasis Area Severity Index (PASI) scores in patients receiving biologics or oral medication [65, 72, 85]. As for GLP-1 RAs specifically, a small study looked at the effects of liraglutide 3 mg daily in 10 PsA and obese patients over a three-month period. The sample was small, but the results were notable – patients received GLP-1 RAs achieved MDA, and both PASI score and pain levels dropped [86]. Another study enrolled 25 individuals with PsO and T2D, randomizing them to acitretin alone or acitretin combined with liraglutide. Patients in the combination group showed improved PASI score and lower IL-17 and IL-23 expression compared to the acitretin-alone subgroup. This finding is interesting as it suggests a direct immunomodulatory effect of liraglutide, independent of weight loss [87]. 

The largest and most methodologically robust study in this area is the TOGETHER-PsA trial (NCT06588296). This was a randomized, 52-week, phase 3b study that compared ixekizumab plus tirzepatide against ixekizumab alone in 271 adults with active PsA and overweight or obesity [88]. The primary endpoint — achieving both ACR50 and at least 10% weight reduction at week 36 — was met by 31.7% of patients in the combination group compared to just 0.8% in the ixekizumab-alone group [88]. Attainment of ACR50 was also more frequent in the combination arm (33.5% vs. 20.4%). What made this result particularly interesting was that the rheumatologic benefit was apparent by week 4 already – before any meaningful weight loss had taken place – which raises the possibility that tirzepatide has direct, anti-inflammatory effects beyond what weight loss can explain [88]. The combination arm also did better in MDA, DAPSA, enthesitis scores, patient-reported outcomes, and cardiometabolic parameters, with a good safety profile [88]. Two important limitations should be acknowledged: tirzepatide targets both GIP and GLP-1 receptors, making it difficult to distinguish how much of the benefit came specifically from the GLP-1 receptor agonism, although the exact incretin-mediated pathway driving weight loss may ultimately be of lesser clinical importance than the overall therapeutic outcome, and non-obese patients were not included [88]. It is therefore currently impossible to draw conclusions about GLP-1 receptor agonism specifically from this trial, and the results should be interpreted as evidence for the broader strategy of targeting obesity in PsA rather than as evidence for GLP-1 RA-specific immunomodulation. Nevertheless, TOGETHER-PsA represents an important step forward in demonstrating that simultaneously targeting inflammation and obesity may yield additive and clinically meaningful benefits in PsD. This approach is also backed by real-world data from a retrospective analysis of two cohorts, confirming that GLP-1 RA therapy significantly improves both metabolic status and outcomes related to PsA [14]. To this end, a parallel trial in patients with PsO (TOGETHER-PsO, NCT06588283) has similarly demonstrated positive outcomes with the combination of ixekizumab and tirzepatide [89]. 

Taken together, GLP-1 RAs may benefit patients with PsD primarily through weight reduction. Whether direct immunomodulatory actions, including modulation of the IL-17 and IL-23 pathways, contribute independently of weight loss remains speculative and has not been demonstrated in adequately designed clinical trials. However, the clinical data remain limited by small sample sizes and heterogeneous study designs. Dedicated randomized controlled trials in PsO and PsA patients – including those who are not obese – will be essential to clarify the direct immunological mechanisms involved and to establish the precise role of GLP-1 RAs in PsD management.

## Discussion: challenges and future perspectives

**Fig. 1:**
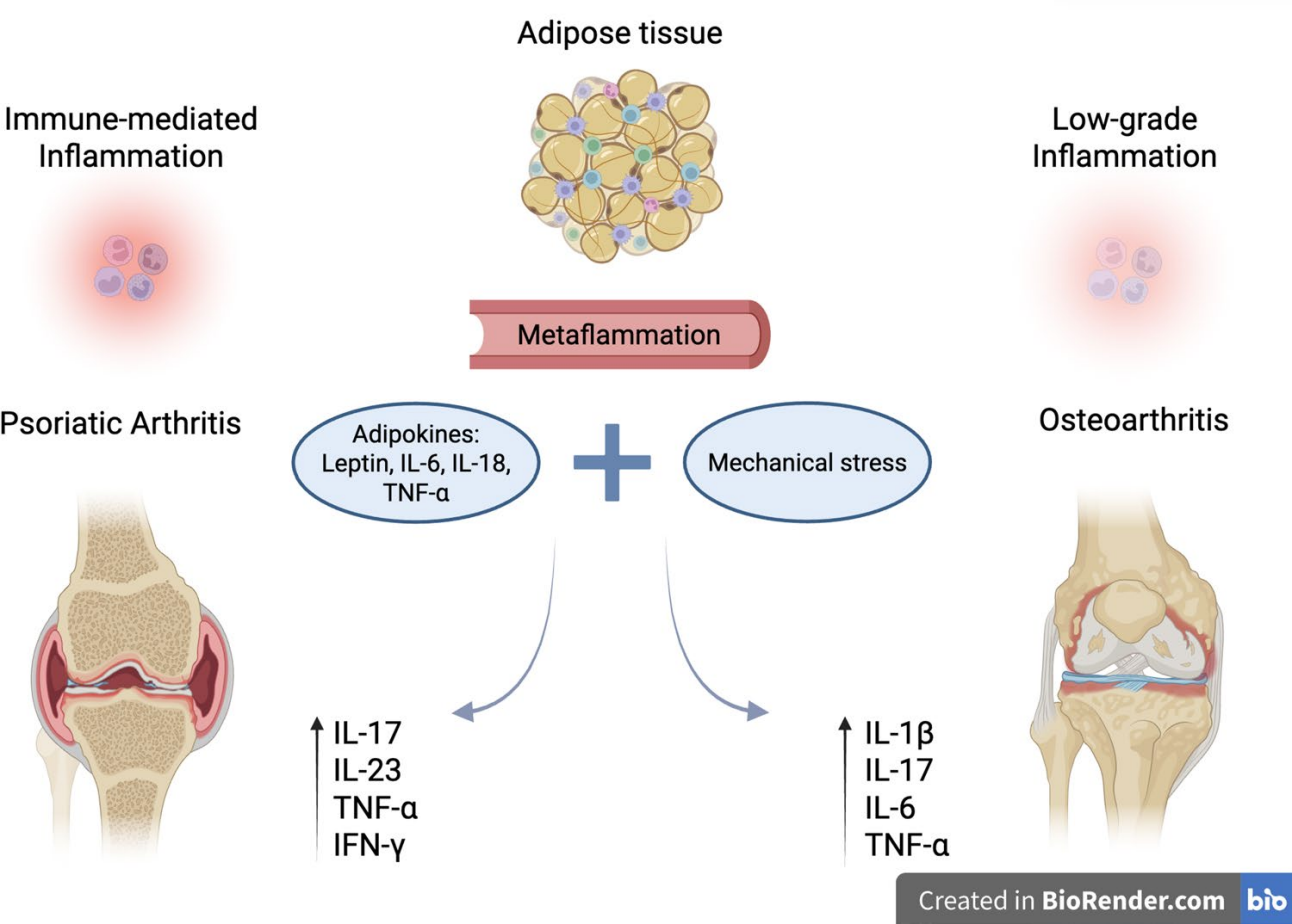
The role of obesity and adipose tissue dysfunction in psoriatic arthritis and osteoarthritis. Dysfunctional adipose tissue drives a state of chronic low-grade systemic inflammation — termed metaflammation — through the release of pro-inflammatory adipokines, while increased mechanical forces are exerted on weight-bearing joints and entheses. These two pathways converge to promote joint inflammation through distinct but partially overlapping mechanisms, driving immune dysregulation and cartilage degradation in psoriatic arthritis and osteoarthritis respectively. Abbreviations: IL, interleukin; TNF-α, tumour necrosis factor-alpha; IFN-γ, interferon-gamma

Obesity rates in developed nations have doubled in the past twenty years and have become a major clinical challenge in RMDs [90]. Instead of just co-occurring with other conditions, obesity actively drives disease progression. It significantly changes the course of RMDs through metaflammation – the chronic, systemic inflammatory response driven by adipose tissue. This process is the main mechanistic connection between obesity and its various musculoskeletal co-morbidities [8, 19], as shown in Fig. 1.

**Fig. 2:**
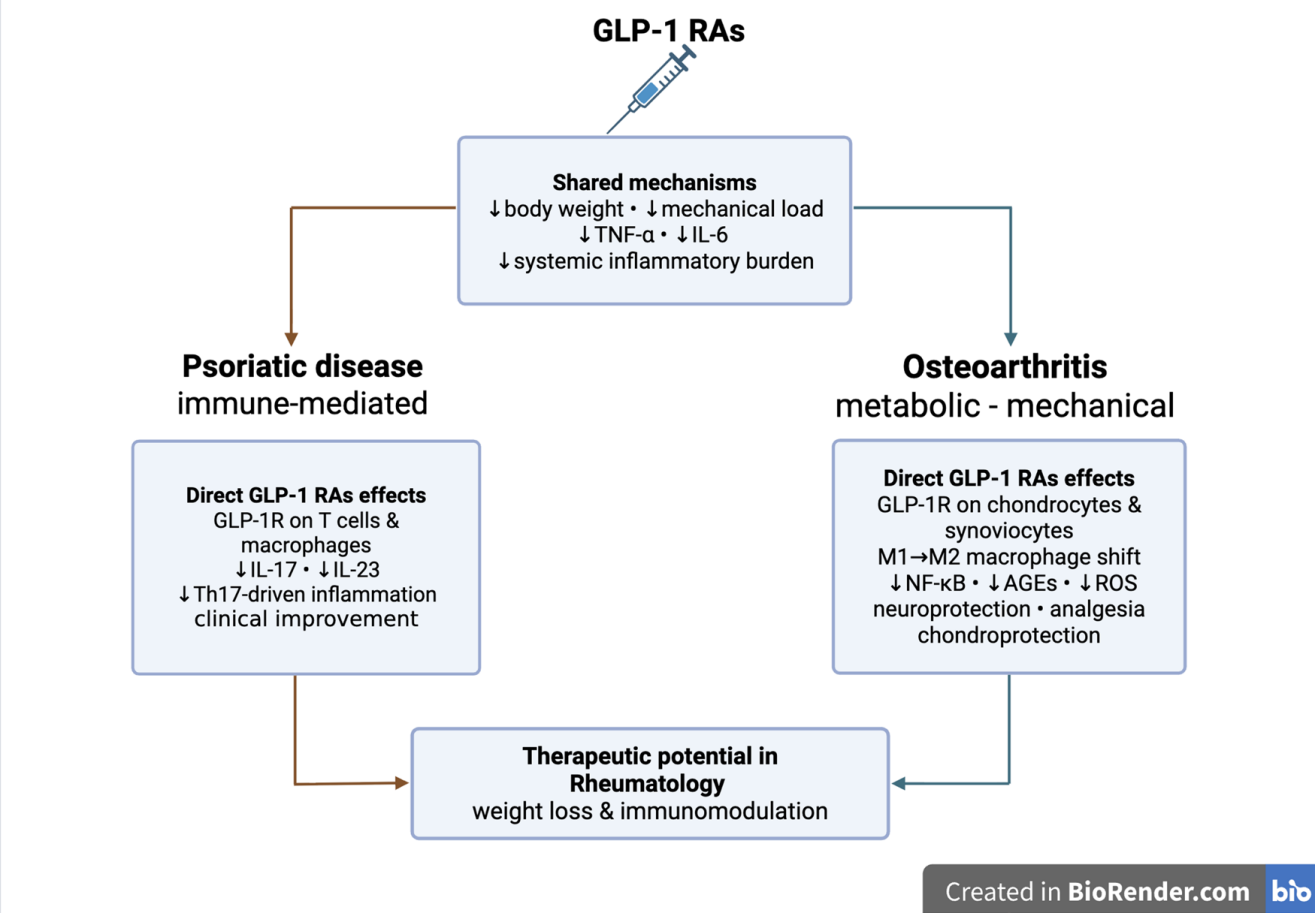
GLP-1 receptor agonists in psoriatic disease and osteoarthritis: proposed mechanisms of action. GLP-1 RAs exert their proposed beneficial effects through shared mechanisms — weight reduction and suppression of systemic inflammation — and disease-specific direct effects mediated through GLP-1R signaling in target tissues. Abbreviations: AGEs, advanced glycation end-products; GLP-1R, glucagon-like peptide-1 receptor; IL, interleukin; MDA, minimal disease activity; NF-κB, nuclear factor kappa B; PASI, Psoriasis Area and Severity Index; ROS, reactive oxygen species; TNF-α, tumour necrosis factor-alpha

Against this background, GLP-1 RAs seem to have a promising role in RMDs as a therapeutic class with potential relevance beyond their established metabolic indications. The evidence reviewed here suggests that their beneficial effects in OA and PsD may operate through two complementary pathways: an indirect pathway mediated by weight reduction, which appears to be the dominant mechanism, which relieves mechanical joint loading, improves cardiometabolic parameters, and reduces the systemic inflammatory burden; and a potentially direct pathway, through GLP-1R-mediated signaling in chondrocytes, synoviocytes, macrophages, and immune cells, which is supported by preclinical data and has not been definitely demonstrated in human studies [5, 7]. These potential actions are depicted in Fig. 2.

In OA, the indirect advantages of weight loss are well established, and the STEP-9 trial has provided meaningful clinical evidence that semaglutide decreases both body weight and OA-associated pain [13]. The expansion of clinical trials to include OA patients without obesity would be particularly informative, as it would allow the direct anti-inflammatory and chondroprotective effects of GLP-1 RAs to be evaluated apart from their weight-reducing effects [38]. It should be mentioned that, as women represent the majority of participants in OA clinical trials due to the higher prevalence of OA among women, this limits our understanding of how GLP-1 RAs may affect men with OA [7]. 

In PsD, the overlap between obesity-driven inflammation and the IL-17/IL-23 axis that dominates psoriatic pathophysiology may provide a biological rationale for GLP-1 RA use, although direct evidence in this context remains limited. It is also notable that, unlike anti-TNF agents, anti-IL-17 and anti-IL-23 therapies tend to maintain their effectiveness more reliably across different weight groups, implying that the pharmacokinetic effects of obesity might vary between biologic classes, which is an important consideration when choosing treatments in obese patients with PsD [76, 77, 79]. The trial results for ixekizumab combined with tirzepatide are promising. However, proper randomized trials with selective GLP-1 RAs in PsA and PsO patients, including individuals who are not obese, are necessary to establish whether this drug class has indeed direct immunomodulatory effects. Another interesting fact is that changes in the gut microbiota, often called dysbiosis, have been linked to the systemic inflammation that connects obesity-induced metabolic dysfunction with PsD development [91, 92]. 

The possibility of a window of opportunity in early PsD deserves particular attention. A large cohort study discovered that a decrease in BMI over ten years was linked to a lower risk of developing PsA in patients with PsO, compared to individuals whose BMI remained stable [93], suggesting that early obesity management may influence the natural history of the disease; whether GLP-1 RAs specifically could contribute to this effect remains hypothetical. This hypothesis, however, is based on observational data and requires prospective validation before any clinical implications can be drawn. Based on the currently available data, GLP-1 RAs cannot yet be formally recommended as part of routine rheumatological management. However, in obese or overweight patients with RMDs, particularly in those with difficult-to-manage or refractory disease or individuals who are at high risk, such as obese patients with PsO who have not yet developed PsA – weight reduction through any effective pharmacological strategy, including GLP-1 RAs, is clinically rational and consistent with existing guidelines advocating for obesity management in this population [90]. Whether GLP-1 RAs offer benefits beyond those of weight loss alone in these patients remains to be established through dedicated clinical trials. It is also noteworthy that other pharmacological agents used in rheumatology, such as leflunomide and apremilast have been shown to help in reducing body weight, although their efficacy may be limited in patients with moderate to severe disease [26]. 

Several other anti-obesity drugs are under investigation. Retatrutide is a new triple receptor agonist that binds to GIP, glucagon, and GLP-1 receptors, currently not approved by the FDA. It shows promising results in body weight reduction and could become a therapeutic choice for obesity management in the future [3, 94]. Whether such agents will demonstrate superior musculoskeletal benefits compared to selective GLP-1 RAs is still unknown.

From a safety perspective, gastrointestinal disturbances – including nausea, vomiting, constipation, and diarrhea – represent the most frequent side effects of GLP-1 RAs. They tend to be mild, vary with dose, and usually fade within the first few weeks of treatment [1]. Particularly, patients with rheumatic diseases may already be treated with conventional DMARDs, such as methotrexate and leflunomide, which are themselves associated with gastrointestinal intolerance; [95] thus, combination with GLP-1 RAs may compound this burden, potentially affecting adherence to both treatments. For patients with RMDs also, the risk of sarcopenia due to weight loss from GLP-1 RA is especially important, as losing lean muscle mass could lead to worse functional outcomes. Hence, adequate protein intake and regular physical exercise should be part of the management when starting these agents [1, 2]. Regarding other safety signals, an increased risk of acute pancreatitis has been reported with GLP-1 RA use, although the causality remains debated [96]. Concerns regarding thyroid C-cell tumors have been raised based on a rodent data but have not been confirmed in human studies [5, 97]. 

This review has the limitations of a narrative review, including potential selection bias in the literature reviewed and the lack of quantitative analysis. Most of the data come from preclinical models, small observational studies and post-hoc analyses, predominantly in obese patients. Whether the findings can be applied to non-obese individuals with OA and PsD remains to be addressed.

In conclusion, GLP-1 RAs are a promising treatment choice for obesity-related musculoskeletal conditions, offering potential advantages for both OA and PsD. Rheumatologists should regard obesity as a modifiable comorbidity and integrate its management into a systematic, holistic approach to patient care [8]. More research is needed, especially in non-obese OA and PsD patients, in order to better understand the exact immunomodulatory mechanisms of GLP-1 RAs and to establish their precise role in the rheumatological therapeutic armamentarium.

## Declarations

### AI assistance

During the preparation of this manuscript, the authors used AI-assisted tools (Claude) to support language editing and structural organization. All AI-generated or AI-assisted content was carefully reviewed, verified and revised by the authors, who take full responsibility for the accuracy and integrity of the final manuscript. No AI tool was used as an author or to generate original scientific conclusions.
